# HIV-1 and opiates modulate miRNA profiles in extracellular vesicles

**DOI:** 10.3389/fimmu.2023.1259998

**Published:** 2023-11-09

**Authors:** Allen Caobi, Jesenia Bonilla, Mario Gomez, Mickensone Andre, Adriana Yndart, Francisco A. Fernandez-Lima, Madhavan P. Nair, Andrea D. Raymond

**Affiliations:** ^1^ Herbert Wertheim College of Medicine at Florida International University, Department of Immunology and Nanomedicine, Miami, FL, United States; ^2^ Florida Memorial University, School of Arts and Sciences, Department of Health and Natural Sciences, Miami Gardens, FL, United States; ^3^ College of Arts, Sciences, and Education at Florida International University, Department of Chemistry, Miami, FL, United States; ^4^ Institute of Neuroimmune Pharmacology in Herbert Wertheim College of Medicine at Florida International University, Miami, FL, United States

**Keywords:** exosomes, HIV, opiates, morphine, miRNA, bioinformatics

## Abstract

Opiate abuse increases the risk of HIV transmission and exacerbates HIV neuropathology by increasing inflammation and modulating immune cell function. Exosomal EVs(xEV) contain miRNAs that may be differentially expressed due to HIV infection or opiate abuse. Here we develop a preliminary exosomal-miRNA biomarker profile of HIV-infected PBMCs in the context of opiate use. PBMCs infected with HIV were treated with increasing dosages of morphine for 72 hours, the culture supernatants were collected, and the exosomes isolated using differential centrifugation. Exosomal miRNAs were extracted, expression levels determined via Nanostring multiplexed microRNA arrays, and analyzed with Webgestalt. The effect of the exosomes on neuronal function was determined by measuring calcium. Preliminary findings show that HIV-1 infection altered the miRNA profile of PBMC-derived EVs concurrently with opiate exposure. MicroRNA, hsa-miR-1246 was up-regulated 12-fold in the presence of morphine, relative to uninfected control. PBMCs infected with HIV-1 MN, an X4-tropic HIV-1 strain and exposed to morphine, displayed a trend which suggests potential synergistic effects between HIV-1 infection and morphine exposure promoting an increase in viral replication. Dose-dependent differences were observed in miRNA expression as a result of opiate exposure. The xEVs derived from PBMCs exposed to morphine or HIV modulated neuronal cell function. SH-SY5Y cells, treated with xEVs derived from ART-treated PBMCs, exhibited increased viability while for SH-SY5Ys exposed to xEVs derived from HIV-1 infected PBMCs viability was decreased compared to the untreated control. Exposing SH-SY5Y to xEVs derived from HIV-infected PBMCs resulted in significant decrease in calcium signaling, relative to treatment with xEVs derived from uninfected PBMCs. Overall, HIV-1 and morphine induced differential miRNA expression in PBMC-derived exosomes, potentially identifying mechanisms of action or novel therapeutic targets involved in opiate use disorder, HIV neuropathology, TNF signaling pathway, NF-κB signaling pathway, autophagy, and apoptosis in context of HIV infection.

## 1 Introduction

Extracellular vesicle (EV) formation occurs in most nucleated hematopoietic cells and is evolutionarily conserved (1, 2). Exosomes are integral to intercellular communication and signal transduction by facilitating the transport of proteins (host and viral proteins) and genetic content [RNA and microRNA (miRNA)] from cell to cell (2, 3). Exosome protein, RNA, and miRNA contents differ depending on the cellular derivation and are modulated in the presence of viral infection (4–6). EVs are classified as one of the following: apoptotic bodies, microvesicles, or exosomes. EVs are classified not just by size but also by the mechanism by which they are produced within a cell. Of interest in this study are exosomes, which are intraluminal vesicles (ILVs) ranging from 30 to 120 nm in diameter, are formed during the maturation of multivesicular bodies within the late-endosome via inward budding of the endosomal membrane, and are released into extracellular space when a multivesicular body (MVB) fuses with the plasma membrane (PM) (1, 5, 7). Microvesicles range between 150 nm and 1 µm in diameter and are primarily generated via shedding/budding from the PM (8). In this study, we primarily focus on an exosome-enriched population of EVs [exosomal extracellular vesicles (xEVs)] consisting primarily of exosomes and a few microvesicles under 220 nm in diameter. HIV is the retrovirus responsible for causing acquired immunodeficiency syndrome (AIDS) (9). HIV is primarily spread via sexual contact across mucosal surfaces but may also be dispersed via maternal–infant exposure and by percutaneous inoculation (10). HIV-1 preferentially infects T cells with a high degree of CD4 surface protein expression and those subsets of T cells expressing CCR5, particularly memory T cells (11, 12).

Similar to CD4+ T cells, CD8+ T cells release exosomes that restrict HIV-1 replication. CD8+ T cell-derived EVs contain an anti-HIV protein moiety that suppresses replication without EV internalization. This indicates that exosome-mediated HIV-1 transcription suppression may comprise an intracellular signaling pathway (13). EVs interact with cellular barriers, the blood–brain barrier (BBB) and the gastrointestinal (GI) barrier, via TLR pathways to promote innate antiviral immunity (14, 15). EVs from HIV-1-infected macrophages and plasma contain HIV-1-derived miRNAs, vmiR88 and vmiR99, promoting macrophage release of TNF-α via TLR8 activation, thus supporting chronic immune activation (16). Exosomal and cellular miRNA profiles are modulated by the HIV-1 Nef protein, affecting both HIV-1 pathogenesis and viral replication (17). EV-encapsulated miRNAs can alter HIV-1 pathology and enhance an infection similar to proteins. There is a large body of evidence demonstrating the relationship between opioid abuse and HIV-1 co-exposure, exacerbating, and hastening the onset of HIV encephalitis among other neurodegenerative conditions.

Opiates have caused medical concern and addiction since the isolation of morphine in 1805, facilitating the treatment of symptoms at the cost of a chronic addiction (18). The USA is currently in the middle of an opioid epidemic, with opioid deaths quadrupling since 1999 (19). Analgesic drugs, such as opioids, play a vital role in pain management, relieving acute severe pain via its action on the µ-opioid receptor, reducing perceived non-cancer pain by 30% (20, 21). With a wide variety of opioid analgesics prescribed to manage chronic pain annually, in the USA, opioids have become the most prescribed class of medication (21, 22). In 2015, the prevalence of opioid misuse in the USA was 21.7%–29.3%, and the prevalence of addiction was 7.8%–11.7% (21). Recently, there has been an increase in worldwide illicit usage of heroin, fentanyl, and prescription opiates (23). This abuse tripled since the 1990s, and opiate overdose deaths have risen by 200% since 2000 in the USA (23). At the center of this crisis, heroin and fentanyl, a short-acting synthetic opiate 100-fold more potent than heroin, have resulted in deaths due to legal prescription overdoses, with an increase in heroin abuse deaths of 26% and fentanyl abuse by 80% (23). Opioid abuse increases risk of HIV transmission and exacerbates HIV neuropathology by increasing inflammation and modulating immune cell function (20, 24–27).

Additionally, opioid abuse has been shown to exacerbate HIV transmission and neuropathology (28–32). Opiate over-consumption and intravenous use significantly contribute to the transmission of HIV among opioid abusers (33–35). Approximately 11% of positive HIV infection diagnoses among adolescents and adults, in 2011, were a result of injection drug users (IDUs) (36). In addition to facilitating viral spread, opioid abuse may modulate HIV-1 pathogenesis. Alone, opioid abuse increases glial activation, induces neuro-inflammatory pathways, and produces changes in the gene expression and conformation of tight junction proteins (25). These effects generate BBB dysfunction, increase BBB permeability, and increase the brain’s vulnerability to peripheral toxins (25). Co-exposure of HIV and opiates enhances HIV neuropathogenesis by elevating monocyte entry into the central nervous system (CNS), increasing adhesion of monocytes and peripheral blood mononuclear cells (PBMCs) to the endothelium, thereby impairing BBB integrity and permeability (37). Given that greater than 50% of all patients positive for HIV-1 develop HIV-1-associated neurocognitive disorder (HAND) and that the cells within the CNS, which are predominantly infected by HIV-1, are microglia, it is probable that infected microglia promote HIV-associated neurotoxicity (38). Although HIV-1 infects astrocytes and results in EV-bound HIV-Nef-derived vascular dysfunction of endothelial cells, HIV-1 is primarily retained and concentrated within the largest cellular reservoir in the brain, infiltrating macrophages and brain microglia (38–44). Given these data, neurotoxic viral proteins and virions are primarily found to be produced from microglia and macrophages. HIV-1-infected microglia have been shown to secrete or induce other neuronal cells to generate neurotoxic factors, which may contribute to HAND (45–50). Therefore, it may be possible for opiates to modulate HIV-1-associated neuropathology by altering microglia and macrophages.

Although neurons are not susceptible to HIV-1 infection directly, neurons suffer injury and sub-lethal stress as a result of HIV-1-infected cells nearby that release HIV proteins and other factors, leading to hyperexcitability (51). For example, the HIV-1 transactivator of transcription (Tat) depolarizes neurons exposed to it, resulting in hyperexcitability and electrophysiological dysfunction via interactions with α-amino-3-hydroxy-5-methyl-4-isoxazolepropionic acid (AMPA) and *N*-methyl-d-aspartic acid (NMDA) receptors; tat also interacts with opiates, activating macrophages and microglia and enhancing the expression of cytokines *in vitro* (52–59). HIV-1 infection and opioid use comorbidity have been linked to an increased severity of HAND (60–63). For this study, morphine was used instead of heroin because is rapidly metabolized in humans to 6-acetylmorphine (6-AM) and then further metabolized to the longer-acting bioactive morphine and morphine conjugates (64).

EVs play dichotomous roles in viral infections and pathology. Not only are EVs critical for intracellular communication, but they may also represent a novel innate antiviral mechanism. Viruses exploit the EV biogenesis pathway to promote viral infection, replication, spread, and pathology. Aside from promoting the associated pathology, viruses use EVs to modulate antiviral immune responses. xEVs are a population of EVs enriched in exosomes, with few if any microvesicles and no apoptotic bodies. In this study, we investigated changes in the miRNA profile of PBMC-derived xEVs, altered because of HIV infection and/or opioid use. This study demonstrated how HIV modulates xEV miRNA cargo and the pathways that may be altered because of this xEV cargo. Morphine was found to differentially modulate miRNA expression of PBMC-derived EVs in the context of HIV infection. Additionally, morphine-treated PBMC-derived xEV miRNAs targeted genes involved in viral replication, apoptosis, and neuronal growth. Overall, this study identified a potential miRNA biomarker profile of opioid use in HIV-1-infected individuals.

## 2 Materials and methods

### 2.1 Cell culture and maintenance

PBMCs were isolated from the blood of anonymous healthy donors using Ficoll-Paque gradient centrifugation and then expanded in a medium containing Roswell Park Memorial Institute (RPMI) 1640 medium with 10% fetal bovine serum (FBS), 100 units/ml of penicillin, and 100 units/ml of streptomycin at 37°C in humidified air with 5% CO_2_. The PBMCs were incubated with phytohemagglutinin-P (PHA-P) for 72 hours at a final concentration of 3 µg/ml. After 72 hours, the PBMCs were centrifuged at 1,500 rpm in a 50-ml conical tube, a pellet was acquired, and the cells were resuspended in 5 ml of fresh media. Polybrene was added to the resuspended cells at a final concentration of 2 µg/ml; the PBMCs were then incubated at 37°C in humidified air with 5% CO_2_ for 30 minutes.

### 2.2 HIV infection

After the cells were incubated in polybrene for 30 minutes, HIV-MN (X4-strain) was added to the samples slated for HIV-1 infection at a concentration of HIV-1 of 20 ng/10 million cells, as previously titrated by p24 ELISA. The HIV-infected cells were incubated at 37°C in humidified air with 5% CO_2_ for 2 hours. The PBMCs were centrifuged again at 1,500 rpm, generating a pellet of PBMCs. The PBMC media were discarded carefully to not disturb the pellet, and the cells were washed with sterile phosphate-buffered saline (PBS) and centrifuged again at 1,500 rpm. Once a pellet was acquired, the cells were placed in 10 ml of fresh complete media and IL-2 at a concentration of 20 U/ml.

### 2.3 Morphine treatment

Cells were infected with HIV at 20 ng per 10e6. Once infected, the cells were treated with various concentrations of morphine diluted in PBS. The morphine concentrations used in this study were 0.02 µM, 0.2 µM, and 2 µM. A negative control of PBS (vehicle) was employed. Morphine was added to cell cultures every 48 hours. Supernatants were collected for exosome isolation, and cells were harvested for RNA isolation. RNA was isolated from exosome pellets and cells using the RNeasy kits (QIAGEN, Valencia, CA, USA) described in Section 2.9. Exosomes were isolated from the supernatants that were collected 7 days post-infection and RNA extracted as described in Section 2.4.

### 2.4 Exosome isolation

Culture media of the PBMCs, an estimated 10 ml, were collected and centrifuged at 300 g for 10 minutes to remove cells. The supernatant was collected, and the resulting pellet was discarded. The supernatant was centrifuged once more at 2,000 *g* for 10 minutes for the removal of dead cells and debris. The pellet was discarded, and the supernatant was collected and centrifuged at 10,000 *g* for 30 minutes to remove smaller debris. The resulting supernatant was sterile filtered via a 0.22-µm filter (Fisher Scientific, Waltham, MA, USA). The filtered supernatant was ultra-centrifuged (UC) (Beckman Coulter, Brea, CA, USA/Optima MAX-XP) at 100,000 *g* for 70 min using a swing bucket rotor (Beckman Coulter/MLS-50). The supernatant was discarded, and 4.5 ml of PBS was then added to the pellet remaining in the UC tube. The UC tubes were ultra-centrifuged again for 70 minutes at 100,000 *g*. Finally, the supernatant was removed, and the pellet was resuspended in 160 µl of PBS.

### 2.5 Characterization of extracellular vesicles

Suspension of EVs (50 µl) isolated via differential ultracentrifugation was added to 950 µl of PBS in a cuvette. EV size and polydispersity index (PDI) were then characterized using dynamic light scattering (DLS) via a Zetasizer (Malvern Panalytical, Malvern, UK). Samples were measured in triplicate.

### 2.6 Digital droplet polymerase chain reaction

HIV-1 replication was measured via digital droplet polymerase chain reaction (ddPCR) (BioRad.com). Isolated RNA (1 µg) was converted to cDNA using the iScript cDNA synthesis kit (BioRad.com, cat#1708890) according to the manufacturer’s instructions. The synthesized cDNA (1 µl) was used for ddPCR. Briefly, cDNA, HIV long terminal repeat (LTR) primers (0.5 µM), and QX200 EvaGreen Supermix (BioRad.com, cat#1864003) were combined with the 20 µl of PCRs. Droplets were formed by combining 70 µl of QX200 Droplet generation oil for EvaGreen (BioRad.com, cat#1864005) with PCRs (20 µl) in a cartridge, and the cartridge was placed in the QX200 droplet generator. The droplet PCRs (40 µl) were placed in a 96-well PCR plate and then placed in a thermocycler; PCR was performed under the following conditions: 1) enzyme reaction 95°C for 5 minutes, for 1 cycle; 2) denaturing at 95°C for 30 seconds for 40 cycles; 4) annealing/extension at 60°C for 1 minute for 40 cycles; and 5) stabilization at 4°C for 1 cycle. PCR products were held at 4°C for 1 hour before analysis. Finally, droplets were analyzed via the QX200 droplet reader (BioRad.com, cat#1864003). Data were reported as the number of copies/20 µl.

*HIV (MN)LTR primers:

Forward: 5′-GGCTAACTAGGGAACCCACTG-3′

Reverse: 5′-GTCAGTGTGGAAAATCTCTAGCAG 3′

### 2.7 NanoString miRNA panels

miRNA was isolated from exosome suspensions using the miRNeasy kit (QIAGEN) according to the manufacturer’s instructions. Isolated miRNA was concentrated via the use of a Vacufuge to at least a concentration of 33.33 ng/µl. The concentrated miRNA was then delivered to NanoString for analysis. The NanoString miRNA Expression Panel was used to detect the expression of over 800 different miRNAs simultaneously.

### 2.8 Exposure of SHSY5Y cells to PBMC-derived xEVs

SHSY5Y cells, acquired from ATCC.org, were cultured on a 96-well assay plate (Corning, New York, NY, USA) for 24 hours at 2.5e5 cells/well in triplicate. After 24 hours, the SH-SY5Y cells were treated with xEVs derived from PBMCs exposed to combinations of HIV and morphine. The protein quantification of EVs isolated from the various PBMC treatments/infections was performed to ascertain the concentration of exosomes per sample. Exosome concentrations were then quantified using 500 ng of protein sample with the Fluorocet exosome quantitation kit. Exosome lysate (1 μg) worth was used to treat the SH-SY5Y cells, per sample. This protein equivalent contained an average concentration of 5.76e7 exosome abundance. After another 24 hours, cell viability and calcium signaling were assayed via CytoQUANT™ XTT Cell Viability Assay (Thermo Fisher) and the Fluo-4 Direct™ Calcium Assay Kit (Thermo Fisher), respectively, as per the manufacturer’s instructions.

### 2.9 RNA isolation

Total RNA was isolated from the infected and non-infected cells and isolated xEVs (exosomal EV pellets derived from treated cells). The RNA was isolated using the RNeasy Plus Mini Kit (QIAGEN) as per the manufacturer’s instructions. Briefly, cells or the exosomal EV pellets were lysed using the RLT lysing solution provided in the RNeasy kit; the lysate was loaded into QIAshredder spin columns and centrifuged for 3 minutes at 10,000 *g*. The QIAshredder flow through was loaded onto the genomic DNA eliminator columns also provided within the kit and centrifuged for 2 minutes at 10,000 *g*. The flow through was mixed with an equal volume of 70% ethanol and then placed in spin columns. The spin columns were washed three times, once with RNA Wash (RW1) (700 μl) and twice with RPE (500 μl) buffers provided in the kit by centrifuging at 10,000 *g* for 1-minute intervals. The RNA was eluted from the spin columns by adding 30 µl of RNAse/DNase-free water and centrifuged for 1 minute at 10,000 *g*. The flow through, now containing the RNAs of interest, was stored at −20°C until use. The isolated RNA was quantified using the Take3 Micro-volume Plate (BioTek, Winooski, VT, USA) and was converted to cDNA using the High-Capacity cDNA Reverse Transcription Kit (Applied Biosystems, Foster City, CA, USA) as per the manufacturer’s instructions. ddPCR was employed to quantify the HIV-1 LTR copy number. EV RNA was acquired by first isolating EVs as described in Section 2.4. The isolated EVs were suspended in 160 μl of PBS, and then RLT lysis buffer (QIAGEN) was added at a 1:1 ratio. This was sufficient to lyse the EVs. RNA of the lysed EVs was quantified using the Take3 system, confirming the successful lysis of the EVs.

### 2.10 Isolation of miRNA

Viral-induced changes in genetic content were analyzed via the NanoString Neuroinflammation Panel. The sample RNA was used in the panel as described per the manufacturer’s instructions. A portion of the generated EVs was used to isolate the miRNA using the miRNeasy Mini Kit (QIAGEN). The lysis solution was also used at a 1:1 ratio with the EV suspension. The miRNA was quantified via the Take3 Micro-Volume Plate (BioTek). MiRNA content was analyzed via the NanoString nCounter Human v3 miRNA panel using the nCounter SPRINT Profiler (NanoString), allowing for simultaneous analysis of over 800 biologically relevant miRNAs. The sample miRNA was used as described per the manufacturer’s instructions. These data were then analyzed using fold change (2^−ΔΔCq^) relative to control.

### 2.11 Statistical and bioinformatic analyses

Experiments were performed in triplicate. Data were analyzed and graphed using GraphPad Prism 6. Statistical significance (p-value <0.05) was determined via ANOVAs (one- and two-way) with *post-hoc* test (Tukey’s test) or (Tukey’s multiple comparisons test). Bioinformatic analysis was performed using the functional enrichment analysis web tool Web-based Gene SeT AnaLysis Toolkit (WebGestalt) to perform Over-Representation Analysis (ORA), determining the Gene Ontology (GO) categories with significantly enriched gene numbers. The enriched Kyoto Encyclopedia of Genes and Genomes (KEGG) pathways were employed via the WebGestalt software.

## 3 Results

### 3.1 The impact of opioid use in the context of HIV-1 infection

**Figure 1:**
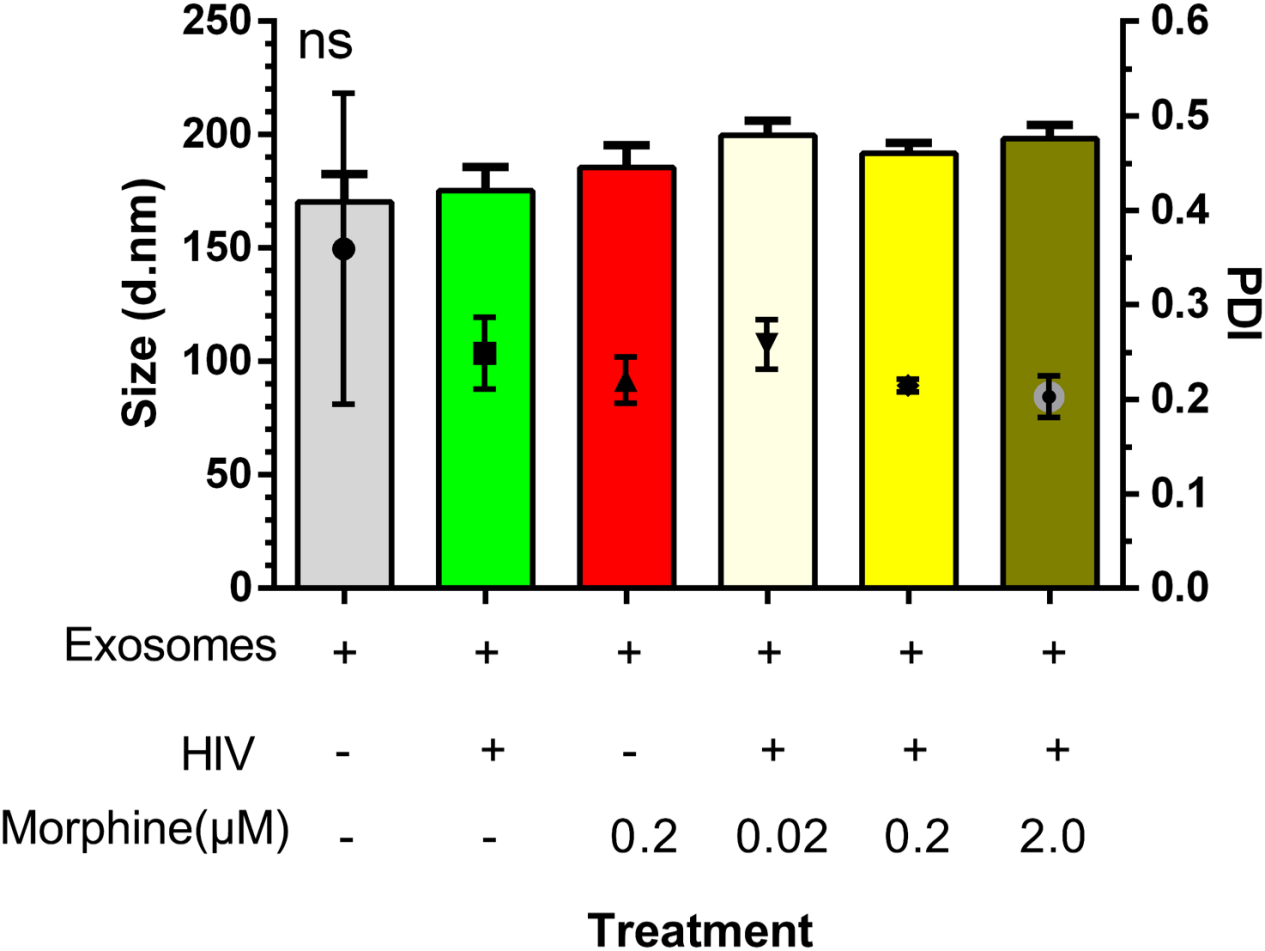
Exosome size was not modulated by viral infection or opioid abuse. Depicted here is a column graph displaying both the average exosome diameter per treatment and the exosome polydispersity index (PDI), as measured via dynamic light scattering (DLS) on a Zetasizer (Malvern Panalytical). ns, not significant.

**Figure 2:**
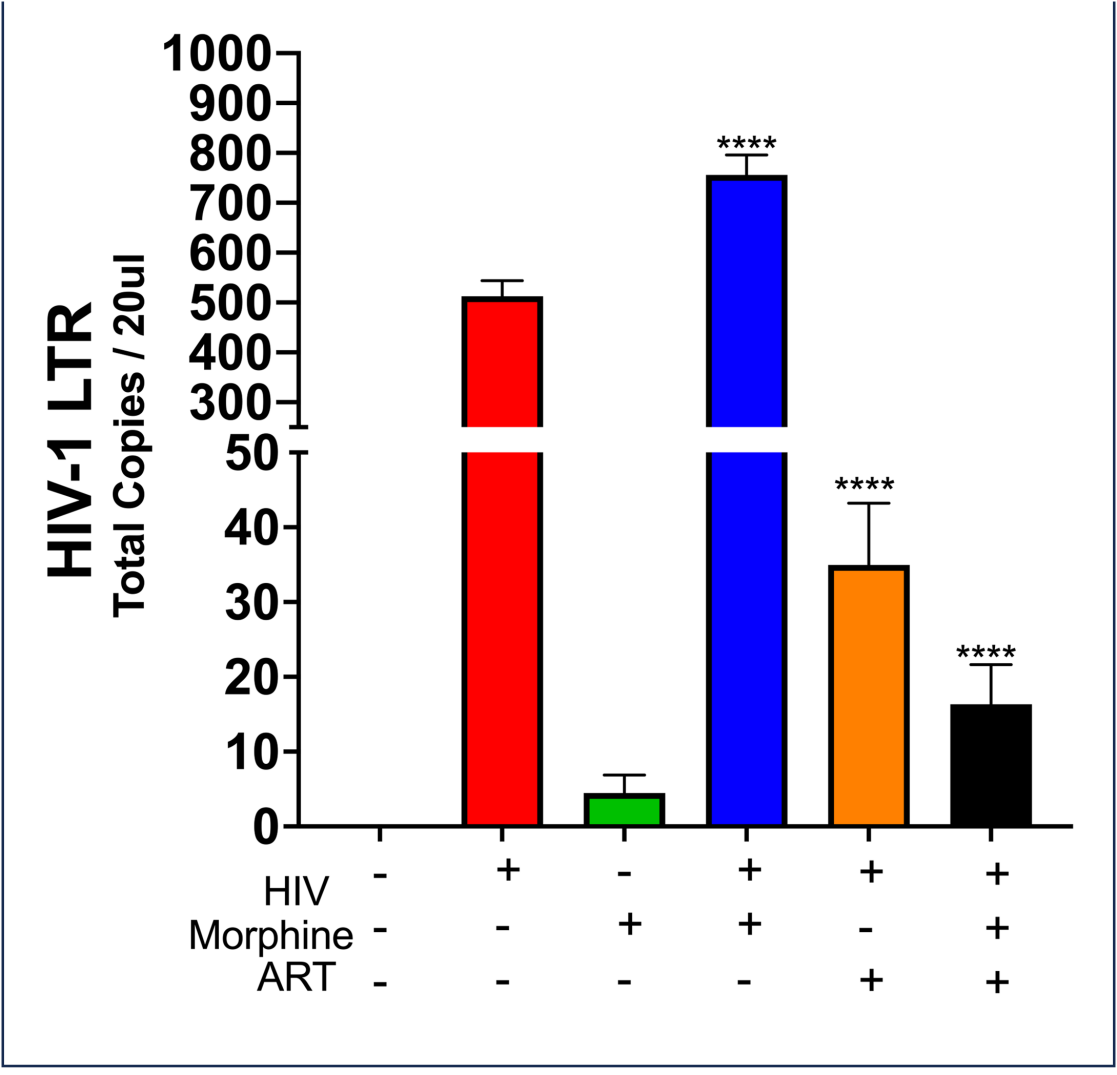
HIV-MN and morphine synergize effects to increase in HIV-MN replication. Selected quantities of RNA (6 ng) used to generate the cDNA used in the measurement of HIV-1 long terminal repeat (LTR) copy numbers post-treatment. Statistical significance determined via one-way ANOVA, with *post-hoc* analysis of Dunnett’s test (****p-value <0.0001).

To determine whether morphine or HIV infection would alter EV size, DLS was used to ascertain both EV diameter and PDI. Neither exosome size nor PDI was found to be modulated by viral infection or opioid abuse (**Figure 1**). To determine the impact of opioid use in the context of HIV-1 infection, the total copy number of the HIV-1 LTR was ascertained per sample via ddPCR. Analysis of the cellular RNA, via ddPCR, confirmed HIV-1 replication in HIV+ samples, in addition to a visible trend suggesting an increase in HIV-1 replication when HIV is in the presence of morphine, displaying a higher copy number, relative to HIV without morphine (**Figure 2**). Samples treated with antiretroviral therapy (ART) presented with diminished HIV-1 viral copy numbers, relative to the untreated infected samples (**Figure 2**). The ART treatment presents a trend demonstrating a decrease in viral replication approximating the values observed in the negative control (**Figure 2**). Interestingly, the ART treatment, composed of tenofovir disoproxil fumarate (TDF) (9.44 µM) and emtricitabine (EMT) (16.17 µM), was used to model how Truvada therapy significantly reduced morphine-enhanced replication of HIV.

### 3.2 HIV-1 infection and opiate exposure alter the miRNA profile of PBMC-derived EVs

**Figure 3:**
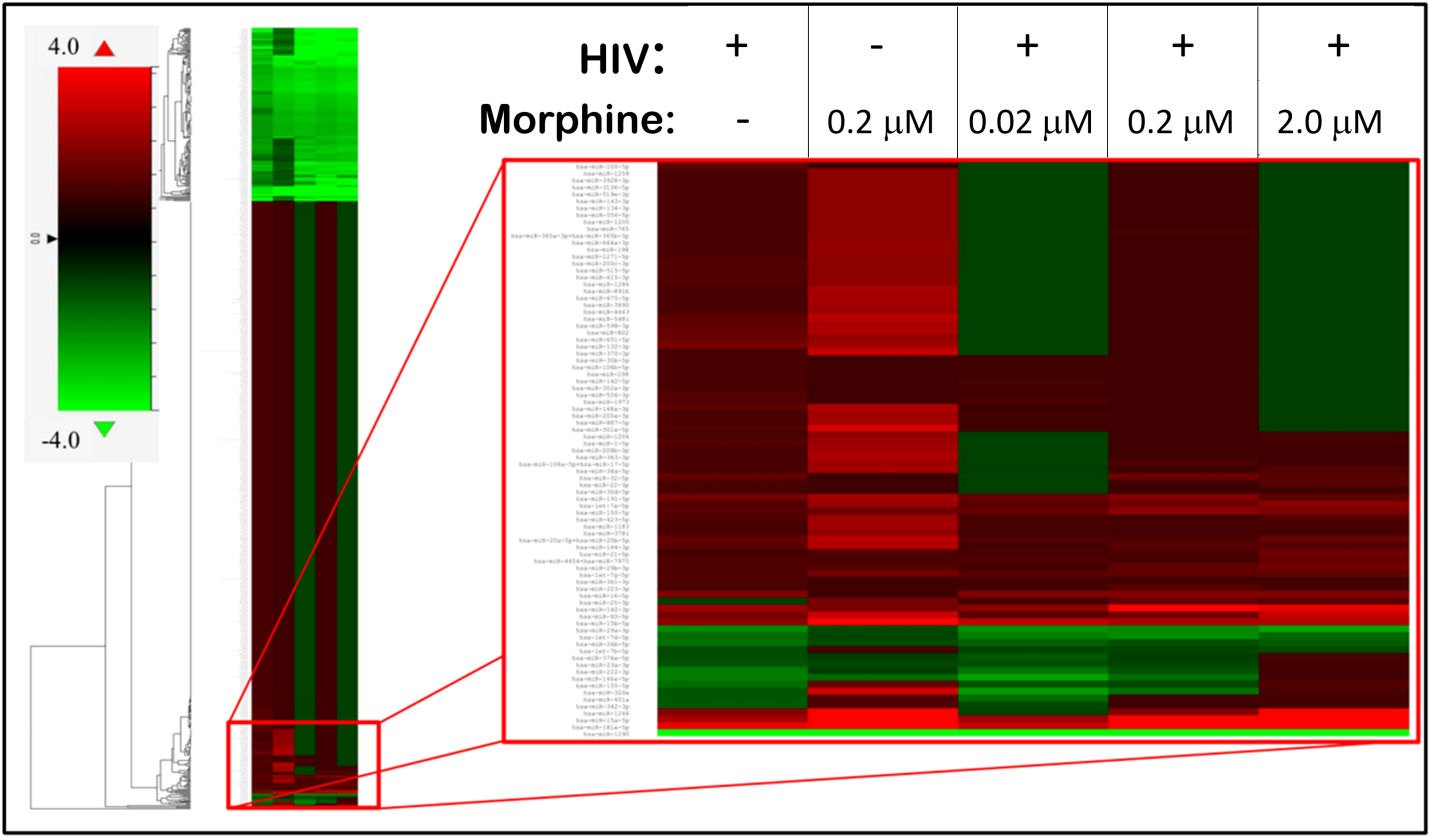
HIV-1 and morphine induced differential miRNA expression in peripheral blood mononuclear cell (PBMC)-derived exosomes, relative to the uninfected control. This is a heat map displaying the HIV- and/or morphine-induced miRNA alterations within PBMC-derived exosomes. On the far left is the fold-change key, which illustrates the difference in fold-change, relative to control, in a color-dependent format. Cells highlighted in red are upregulated, whereas cells presented in green are downregulated.

**Figure 4:**
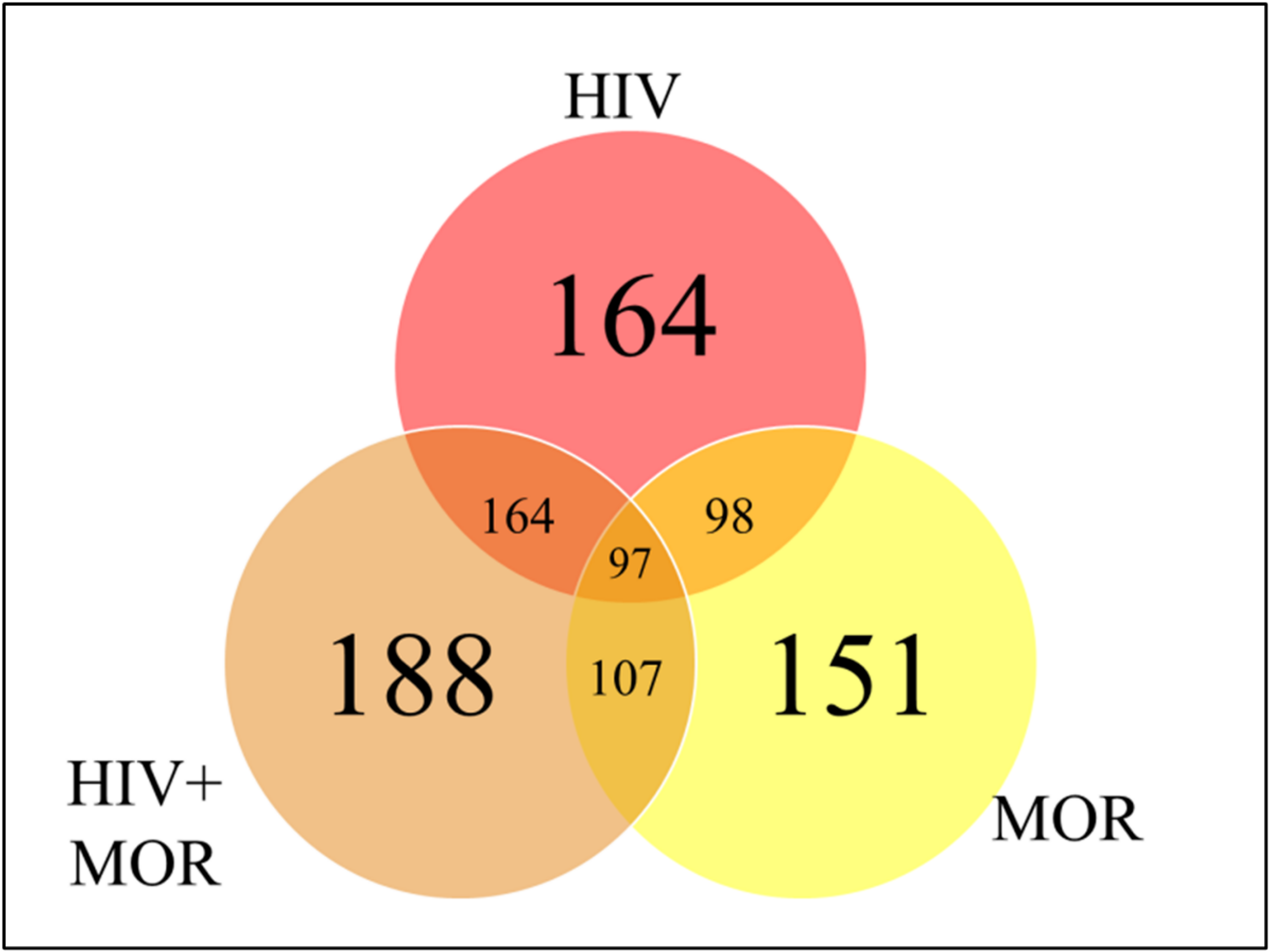
MiRNAs from HIV and/or morphine-treated peripheral blood mononuclear cell (PBMC)-derived extracellular vesicles (EVs) are significantly differentially expressed across treatment groups. Shown here is a Venn diagram depicting the miRNAs that are shared or in common between the varying treatment groups, HIV, HIV, and morphine (HIV+MOR) (0.2 µM), and morphine alone (MOR) (0.2 µM), all relative to the uninfected control.

To determine whether the miRNA profile of PBMC-derived EVs was altered as a result of HIV infection or opioid use, a heat map was generated using the miRNA panel data acquired from NanoString. Infection with HIV-MN strain and/or morphine treatment resulted in the modulation of the miRNA profile of the PBMC-derived EVs, thus presenting a unique miRNA profile that seems to be treatment-dependent due to the differential expression of miRNA (**Figure 3**). The lower portion of the heat map was selected for bioinformatic analysis for its highly varied region (**Figure 3**). To ascertain shared groups of miRNAs among the different treatment groups (**Figure 3**), heat map data were analyzed further. Analysis of the heat map, which consisted of a total of 797 miRNAs, yielded that HIV significantly modulates 164 miRNAs, morphine significantly alters the expression of 151 miRNAs, and the combination of both HIV-1 and morphine modifies 188 miRNAs (**Figure 4**). Of note, all 164 miRNAs that HIV alters are the exact same miRNAs also altered by the combination of HIV and morphine (**Figure 4**). This means that there are no miRNAs that HIV uniquely modulates when compared to HIV and morphine, as all 164 miRNAs are also differentially expressed in HIV and morphine (HIV+MOR) (**Figure 4**). There are 24 miRNAs unique to HIV+MOR when compared to just HIV (**Figure 4**). However, there are 66 miRNAs unique to HIV, when compared to morphine treatment alone (MOR) (**Figure 4**). There are 107 miRNAs that are modulated as a result of either HIV+MOR or MOR alone (**Figure 4**). Finally, there are 97 miRNAs that all three treatment groups shared (**Figure 4**).

### 3.3 Opiate dose-dependent differences in miRNA expression

**Figure 5:**
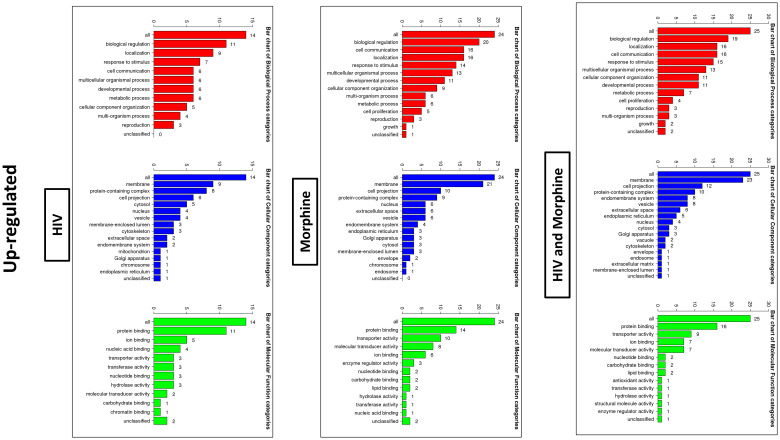
Bioinformatics: GO Slim summary of upregulated miRNAs. Biological Process, Cellular Component, and Molecular Function categories are represented by red, blue, and green bars, respectively. The height of the bar represents the number of IDs in the user list and the category. The numbers on top are the number of target DNAs entered that are in that category.

**Figure 6:**
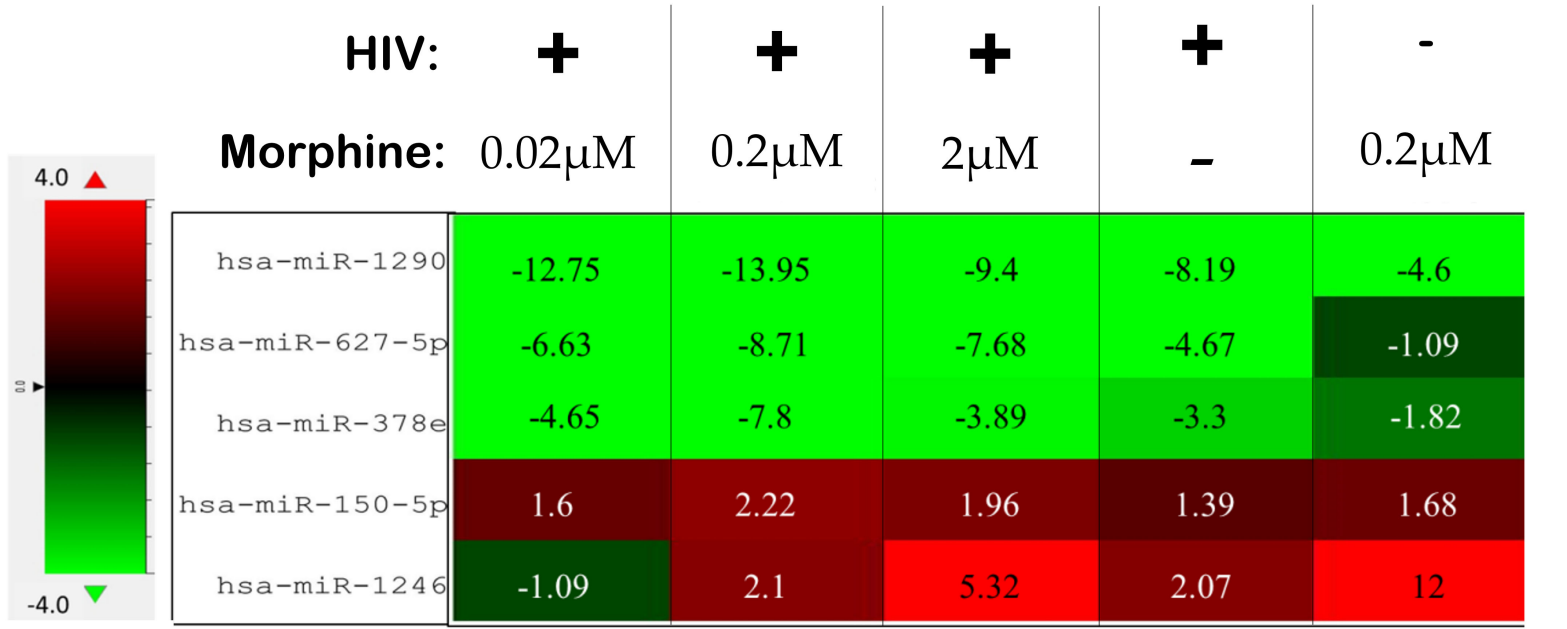
Differentially altered expression of miRNAs, which target key genes involved in viral replication, apoptosis, and neuronal cells. The numbers listed within each cell are the fold change as a result of the listed treatment/infection relative to the uninfected and untreated control. The uninfected and untreated control is the set of exosomes derived from uninfected and untreated peripheral blood mononuclear cells (PBMCs).

**Figure 7:**
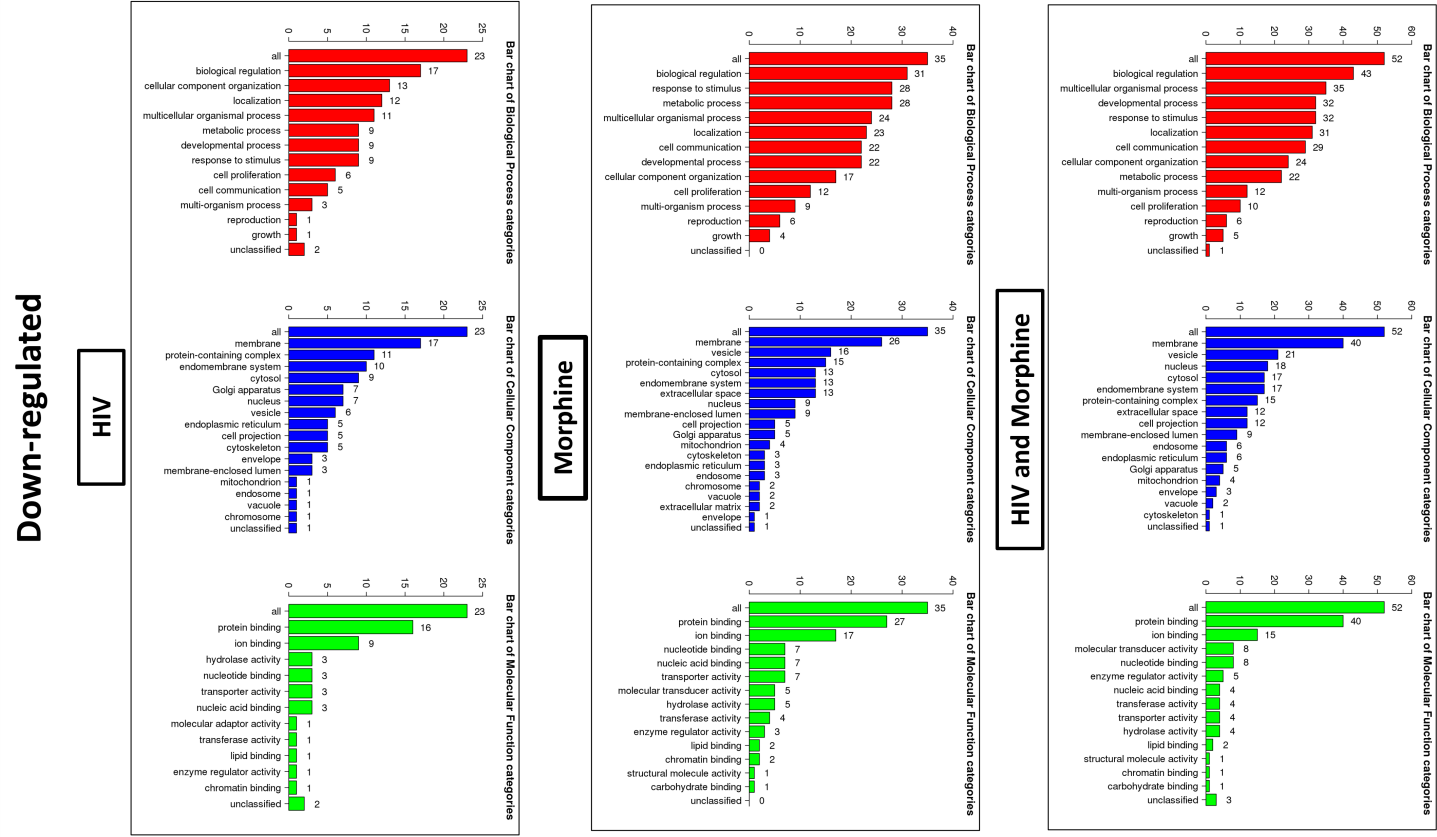
Bioinformatics: GO Slim summary of downregulated miRNAs. Biological Process, Cellular Component, and Molecular Function categories are represented by red, blue, and green bars, respectively. The height of the bar represents the number of IDs in the user list and the category. The numbers on top are the number of target DNAs entered that are in that category.

In order to ascertain if there were any significant differences, among the treatment groups, in the expression of EV-bound miRNAs, miRNAs were selected based on the perceived fold-change differences in expression as a result of the varied treatment groups (**Table 1**). From this dataset (**Table 1**), the miRNAs either targeting genes of interest or presenting with a great difference in expression, as a result of the exposed treatment, were selected for potential miRNA mimic or CRISPR experiments downstream (**Figure 5**). Hsa-miR-1246 presents with a 12-fold upregulation when in the presence of morphine solely (**Figure 5**). However, this drops when in combination with HIV, reducing as low as −1.09-fold change for the HIV+MOR (0.02 µM) treatment. On the other end of the spectrum, hsa-miR-1290 presents large fold-change modulations; however, they are all negative (**Figure 6**). The MOR treatment alone resulted in a −4.6-fold decrease of hsa-miR-1290. However, the combination of HIV+MOR at 0.2 µM dropped miR-1290 expression even lower, down to −13.95-fold change relative to control (**Figure 6**). According to the GO Slim summary, across all treatment groups, upregulated miRNAs primarily target genes involved in biological regulation and response to stimulus (**Figure 5**). MiRNAs targeting the membrane were among the most modulated due to HIV and/or MOR treatment (**Figures 5**, **7**). Protein and ion binding were among the two molecular functions most targeted by the downregulated miRNAs altered in HIV-1 and/or morphine treatment (**Figure 7**).

**Table 1:** HIV differentially modulates miRNA expression of PBMC-derived EVs in the context of opiate exposure.

Probe name	HIV *vs.* NC fold change	HIV+MOR (0.2 μM) *vs.* NC	MOR (0.2 μM) *vs.* NC
hsa-miR-1290	**−8.19**	**−13.95**	**−4.6**
hsa-miR-1253	**−5.6**	**−7.21**	**−2.11**
hsa-miR-371a-5p	**−5.36**	**−8.92**	**−2.15**
hsa-miR-502-5p	**−4.85**	**−5.56**	**−5.53**
hsa-miR-603	**−4.85**	**−7.87**	**−2.05**
hsa-miR-4536-5p	**−4.75**	**−5.45**	**−1.9**
hsa-miR-627-5p	**−4.67**	**−8.71**	**−1.09**
hsa-miR-570-3p	**−4.66**	**−5.35**	**−5.32**
hsa-miR-548aa+hsa-miR-548t-3p	**−4.48**	**−5.14**	**−2.3**
hsa-miR-4531	**−4.39**	**−5.03**	**−5**
hsa-miR-411-5p	**−4.12**	**−5.08**	**−1.64**
hsa-miR-644a	**−4.02**	**−4.62**	**−1.52**
hsa-miR-1246	**2.07**	**2.1**	**12**
hsa-miR-15b-5p	**2.24**	**2.72**	**4**
hsa-miR-142-3p	**2.42**	**4**	**1.89**
hsa-miR-15a-5p	**2.62**	**4.22**	**4.33**
hsa-miR-181a-5p	**3.65**	**4.09**	**4.98**

NC is the set of exosomes derived from uninfected and untreated PBMCs.

PBMC, peripheral blood mononuclear cell; EVs, extracellular vesicles.

To determine the pathways that would most likely be affected as a result of the transport and release of EVs derived from morphine and/or HIV infection of PBMCs, enriched pathway data were acquired through WebGestalt’s Over-Representation Analysis (**Tables 2**–**4**). The gene targets of the upregulated or downregulated miRNAs were acquired. Genes of interest were selected from the list and subsequently used in WebGestalt’s Over-Representation Analysis.

**Table 2:** Summary of gene sets: HIV relative to uninfected and untreated control.

	Upregulated miRNAs	HIV				
Gene set	Description	Size	Expect	Ratio	p-Value	FDR
**hsa02168**	Herpes simplex infection	185	0.099076	20.186	0.0035431	1
**hsa04710**	Circadian rhythm	31	0.016602	60.234	0.016502	1
**hsa04520**	Adherens junction	72	0.038559	25.934	0.038013	1
**hsa04725**	Cholinergic synapse	112	0.059981	16.672	0.058657	1
**hsa04728**	Dopaminergic synapse	131	0.070157	14.254	0.068346	1
**hsa04514**	Cell adhesion molecules (CAMs)	144	0.077119	12.967	0.074932	1
**hsa04080**	Neuroactive ligand–receptor interaction	277	0.14835	6.7410	0.14032	1
	Downregulated miRNAs					
Gene set	Description	Size	Expect	Ratio	p-Value	FDR
**R-HSA-112316**	Neuronal system	368	0.48816	8.1941	0.0011025	1
**R-HSA-204626**	Hypusine synthesis from Eif5a-LYSINE	4	0.0053060	188.46	0.0052962	1
**R-HSA-6794362**	Protein–protein interactions at synapses	87	0.11541	17.330	0.0057319	1
**R-HSA-1296071**	Potassium channels	99	0.13132	15.229	0.0073654	1
**R-HSA-199991**	Membrane trafficking	628	0.83305	4.8016	0.0076943	1
**R-HSA-5653656**	Vesicle-mediated transport	667	0.88478	4.5209	0.0095028	1
**R-HSA-1296052**	Ca^2+^-activated K^+^ channels	9	0.011939	83.762	0.011880	1
**R-HSA-8963888**	Chylomicron assembly	9	0.011939	83.762	0.011880	1
**R-HSA-8963898**	Plasma lipoprotein assembly	18	0.023877	41.881	0.023629	1
**R-HSA-388844**	Receptor-type tyrosine-protein phosphatases	20	0.026530	37.693	0.026222	1

The table concisely summarizes the enriched functional categories with their statistics. Ratio of enrichment equals the number of observed divided by the number of expected genes from each GO or KEGG category in the gene list.

GO, Gene Ontology; KEGG, Kyoto Encyclopedia of Genes and Genomes; FDR, false discovery rate.

**Table 3:** Summary of gene sets: morphine (0.2 μM) relative to uninfected and untreated control.

	Upregulated miRNAs	Morphine				
Gene set	Description	Size	Expect	Ratio	p-Value	FDR
**hsa04672**	Intestinal immune network for IgA production	49	0.091846	32.663	0.000091816	0.030208
**hsa04924**	Renin secretion	65	0.12184	16.415	0.0063441	0.52354
**hsa04742**	Taste transduction	83	0.15558	12.855	0.010181	0.52354
**hsa05166**	Human T-cell leukemia virus 1 infection	255	0.47798	6.2765	0.010827	0.52354
**hsa05032**	Morphine addiction	91	0.17057	11.725	0.012148	0.52354
**hsa04064**	NF-kappa-B signaling pathway	95	0.17807	11.232	0.013189	0.52354
**hsa04080**	Neuroactive ligand–receptor interaction	277	0.51921	5.7780	0.013555	0.52354
**hsa04660**	T-cell receptor signaling pathway	101	0.18932	10.564	0.014821	0.52354
**hsa04625**	C-type lectin receptor signaling pathway	104	0.19494	10.260	0.015669	0.52354
**hsa04060**	Cytokine–cytokine receptor interaction	294	0.55108	5.4439	0.015913	0.52354
	Downregulated miRNAs					
Gene set	Description	Size	Expect	Ratio	p-Value	FDR
**hsa04917**	Prolactin signaling pathway	70	0.23430	17.072	0.000077145	0.013451
**hsa04550**	Signaling pathways regulating pluripotency of stem cells	139	0.46526	10.747	0.000081767	0.013451
**hsa04380**	Osteoclast differentiation	128	0.42844	9.3362	0.00078749	0.072406
**hsa05213**	Endometrial cancer	58	0.19414	15.453	0.00090536	0.072406
**hsa05223**	Non-small cell lung cancer	66	0.22091	13.58	0.0013192	0.072406
**hsa05224**	Breast cancer	147	0.49203	8.1295	0.0013205	0.072406
**hsa04218**	Cellular senescence	160	0.53555	7.469	0.0018058	0.084870
**hsa01521**	EGFR tyrosine kinase inhibitor resistance	79	0.26443	11.345	0.0022152	0.091102
**hsa04933**	AGE-RAGE signaling pathway in diabetic complications	99	0.33137	9.0533	0.0042044	0.15369
**hsa04668**	TNF signaling pathway	110	0.36819	8.148	0.0056467	0.16400

The table concisely summarizes the enriched functional categories with their statistics. Ratio of enrichment equals the number of observed divided by the number of expected genes from each GO or KEGG category in the gene list.

GO, Gene Ontology; KEGG, Kyoto Encyclopedia of Genes and Genomes; FDR, false discovery rate.

**Table 4:** Summary of gene sets: HIV+morphine (0.2 μM) relative to uninfected and untreated control.

	Upregulated miRNAs	HIV and morphine				
Gene set	Description	Size	Expect	Ratio	p-Value	FDR
**hsa04672**	Intestinal immune network for IgA production	49	0.11809	16.937	0.0060330	1
**hsa05110**	*Vibrio cholerae* infection	50	0.1205	16.598	0.0062755	1
**hsa04520**	Adherens junction	72	0.17352	11.526	0.012690	1
**hsa04742**	Taste transduction	83	0.20003	9.9987	0.016634	1
**hsa05032**	Morphine addiction	91	0.21931	9.1197	0.019790	1
**hsa04080**	Neuroactive ligand–receptor interaction	277	0.66756	4.494	0.027249	1
**hsa04060**	Cytokine–cytokine receptor interaction	294	0.70853	4.2341	0.031776	1
**hsa04514**	Cell adhesion molecules (CAMs)	144	0.34703	5.7631	0.046162	1
**hsa04130**	SNARE interactions in vesicular transport	34	0.081939	12.204	0.078930	1
**hsa05330**	Allograft rejection	38	0.091579	10.920	0.087819	1
	Downregulated miRNAs					
Gene set	Description	Size	Expect	Ratio	p-Value	FDR
**hsa04514**	Cell adhesion molecules (CAMs)	144	0.48199	12.448	0.0000060576	0.0019930
**hsa04668**	TNF signaling pathway	110	0.36819	8.148	0.0056467	0.92888
**hsa04010**	MAPK signaling pathway	295	0.98741	4.051	0.015655	1
**hsa04360**	Axon guidance	175	0.58575	5.1216	0.019914	1
**hsa04912**	GnRH signaling pathway	93	0.31129	6.4249	0.038219	1
**hsa04640**	Hematopoietic cell lineage	97	0.32468	6.16	0.041261	1
**hsa05142**	Chagas disease (American trypanosomiasis)	102	0.34141	5.858	0.045188	1
**hsa04620**	Toll-like receptor signaling pathway	104	0.34811	5.7454	0.046797	1
**hsa04722**	Neurotrophin signaling pathway	119	0.39831	5.0212	0.059516	1
**hsa04060**	Cytokine–cytokine receptor interaction	294	0.98407	3.0486	0.073329	1

The table concisely summarizes the enriched functional categories with their statistics. Ratio of enrichment equals the number of observed divided by the number of expected genes from each GO or KEGG category in the gene list.

GO, Gene Ontology; KEGG, Kyoto Encyclopedia of Genes and Genomes; FDR, false discovery rate.

Here, the targets of the miRNAs, DNA, are grouped by gene set (**Tables 2**–**4**). A gene set is a group of genes that share a particular function. This function could be a pathway, a trait such as morphine addiction, or a molecular function such as renin secretion. Relative to the uninfected and untreated control, HIV-induced changes in PBMC-derived EV cargo yield miRNAs targeting: the cholinergic/dopaminergic synapse, neuroactive ligand–receptor interaction, the neuronal system, K^+^ channels, membrane trafficking, vesicle-mediated transport, apoptosis, and RNA degradation (**Table 2**). However, morphine (0.2 µM) modulates EV cargo, such that EV-bound miRNAs target morphine addiction, NF-κ-beta signaling pathway, T-cell receptor signaling pathway, neuroactive ligand–receptor interaction, stem cell pluripotency signaling pathways, TNF signaling pathway, and cellular senescence (**Table 3**). The combination of HIV and morphine (0.2 µM) alters PBMC-derived EV content to yield EVs transporting miRNAs targeting morphine addiction, neuroactive ligand–receptor interaction, cytokine–cytokine receptor interaction, SNARE interactions in vesicular transport, axon guidance, TNF signaling pathway, TLR-signaling pathway, and neurotropin signaling pathway (**Table 4**). Together, these findings suggest that morphine exposure could directly modulate the exosomes released by peripheral immune cells.

### 3.4 PBMC-derived xEVs modulated neuronal cell function

**Figure 8:**
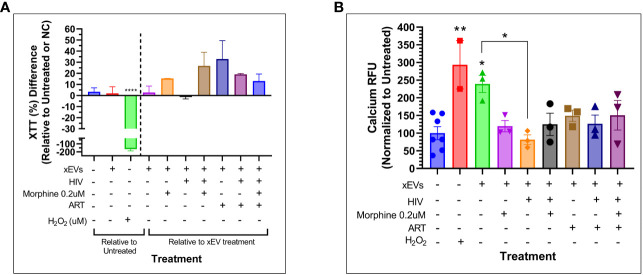
Cytotoxicity and calcium signaling study of SH-SY5Y cells exposed to peripheral blood mononuclear cell (PBMC)-derived exosomal extracellular vesicles (xEVs) after 24 hours of exposure to the xEVs. **(A)** SH-SY5Y cell viability as measured by Tyquan™ XTT Cell Viability Assay (Thermo Fisher). Data shown as percent (%) difference relative to the SH-SY5Y cells exposed to xEVs derived from uninfected and untreated PBMCs (purple column), right of the dashed line, and relative to the untreated PBMCs, left of the dashed line. **(B)** Calcium signaling as measured by the Fluo-4 Direct™ Calcium Assay Kit (Thermo Fisher). Data shown are normalized to the untreated. The H2O2 concentration used in both assays was 3.2 mM.

To determine whether PBMC-derived xEVs impact neuronal cell function, in the context of HIV infection and opioid abuse, SH-SY5Y cells were directly exposed to xEVs derived from PBMCs that were infected with HIV or exposed to morphine, and cell viability and calcium signaling were measured. After exposure to xEVs derived from ART-treated PBMCs, SH-SY5Y exhibited an increasing trend in viability, whereas neuronal cells treated with xEVs derived from HIV-infected PBMCs presented with lower viability, relative to the xEVs derived from ART-treated PBMCs (**Figure 8A**). Additionally, exposure of SH-SY5Y cells to xEVs from morphine-treated PBMCs, with or without HIV, resulted in an increase in cell viability (**Figure 8A**). The impact of SH-SY5Y morphine exposure to PBMC-derived xEVs on calcium signaling was assayed. Exposure of SH-SY5Y cells to xEVs derived from HIV-infected PBMCs significantly reduced calcium signaling (**Figure 8B**). This drop in calcium levels induced by exposure to xEVs derived from HIV-infected PBMCs demonstrates a greater than −150% decrease in calcium signaling, relative to cells exposed to exosomes from healthy PBMCs. Taken together, the data show that the effect of exosomes on neuronal activation is treatment dependent.

## 4 Discussion

No changes in exosome diameter were observed as a result of either HIV infection or morphine treatment. An increase in exosome size could have meant a lower concentration of exosomes or an increase in other EV subtypes such as microvesicles. Treatment with ART Truvada successfully reduced viral replication, even in the presence of morphine, which enhanced HIV replication. PBMCs exposed to HIV, morphine, or ART released exosomes that do not affect calcium levels in neurons, unlike control cell-derived exosomes. Calcium functions as both an intracellular messenger and a charge carrier (65). Calcium signals regulate various developmental processes and have a key role in apoptosis, neurotransmitter release, and membrane excitability (66, 67). Additionally, given its role in synaptic activity and depolarizing signal transmission, calcium signaling is crucial to neurons (68). Thus, modulation of calcium signaling would be indicative of an effect on neuronal cell function. SH-SY5Y viability was quantified. The significance of calcium in neuronal cell signaling and function highlights the potential impact of the changes in exosome content released by peripheral cells due to HIV, morphine, or ART on neuronal function. These changes in PBMC-derived exosome cargo can potentially impair neuronal function, potentially contributing to HIV neuropathology in people living with HIV (PLWHs) who use opiates.

The NanoString miRNA panel data may identify miRNA biomarker profiles that differentiate HIV-1-infected individuals who abuse opiates. Of the miRNAs of interest, hsa-miR-1290 has been reported to contribute to HIV-1 latency, targeting the HIV-1 3′ UTR and suppressing viral expression. Significant downregulation of this miRNA could result in increased viral replication. miR-1290 affects pathways such as binding and uptake of ligands by scavenger receptors and phagocytosis of microbes, Akt signaling, STAT3 pathway, TNF superfamily, and NF-kappa-B signaling. Also of note, the mu-opioid receptor gene has been previously found to be expressed in human immune cells (69–71). However, there is some controversy regarding the presence and expression of the mu-opioid receptor on PBMCs, which must be considered (72–74).

Hsa-miR-627-5p is of interest primarily because of both the pathways associated with this miR and the potential synergistic downregulation observed. The miR-627-5p gene targets are involved in Toll-like receptor-4 (TLR4), interleukin-2, and TNF signaling, activation of cAMP-dependent PKA, and neuropathic pain-signaling in dorsal horn neurons. Other targets of miR-627 include genes that are integral to axon guidance, neurotransmitter clearance in the synaptic cleft, Rho GTPase cycle, and signal transduction and signaling by Rho GTPases. This strongly suggests that miR627 expression levels could correlate with neurological deficits. Similarly, hsa-miR-378e is also of interest because of potential synergistic effects driving downregulation and the pathways that may be altered as a result of miRNA-mediated gene silencing. Pathways modulated by miR-378e may include EVs targeting nuclear receptor transcription pathway, p38 MAPK signaling pathway, gene expression, innate immune system, neutrophil degranulation, membrane trafficking, vesicle-mediated transport, AMPK signaling pathway, muscle/cardiac contraction, neuroactive ligand–receptor interaction, signaling by GPCR, and peptide ligand-binding receptors. Although there does not appear to be any significant difference between treatment groups, regarding hsa-miR-150-5p, this particular miRNA has been reported in multiple studies to be modulated by patients with highly active antiretroviral therapy (HAART) resistance who exhibited downregulation in miR150 expression, which has also been demonstrated to target the HIV-1 RNA genome limiting viral expression. However, hsa-miR-1246 is primarily of interest as a result of the experimental data acquired, although it has been previously reported to be upregulated 7.4-fold in the HIV+ group relative to the control group.

Overall, HIV-1 and morphine induced differential miRNA expression in PBMC-derived exosomes, which may potentially impact multiple pathways by altering EV-bound miRNA cargo, resulting in the modulation of genes involved in the neuronal system, cell–cell communication, TNF signaling pathway, morphine addiction, NF-κ-beta signaling pathway, autophagy, and apoptosis. The exosomal miR profile could be developed as a biomarker or a monitoring system for cellular function and health in PLWHs using opiates or just for opioid users. These profiles could also potentially lead to novel therapeutic targets of HIV pathology and opioid use disorder.

## Data availability statement

The raw data supporting the conclusions of this article will be made available by the authors, without undue reservation.

## Ethics statement

Ethical approval was not required for the studies on humans in accordance with the local legislation and institutional requirements because only commercially available established cell lines were used.

## Author contributions

AC: Conceptualization, Formal Analysis, Investigation, Writing – review & editing, Data curation, Methodology, Writing – original draft. JB: Data curation, Investigation, Writing – review & editing, Formal Analysis, Methodology. MG: Data curation, Formal Analysis, Investigation, Methodology, Software, Writing – review & editing. MA: Investigation, Writing – review & editing, Data curation. AY: Methodology, Writing – review & editing. FF-L: Formal Analysis, Methodology, Writing – review & editing. MN: Formal Analysis, Supervision, Writing – review & editing. AR: Conceptualization, Formal Analysis, Funding acquisition, Investigation, Resources, Supervision, Writing – review & editing.
