# Supplementary material for: HIV-1 and opiates modulate miRNA profiles in extracellular vesicles
Source: Front Immunol. 2023 Nov 9;14:1259998. doi: 10.3389/fimmu.2023.1259998 (PMC10666642; doi:10.3389/fimmu.2023.1259998)
Supplement: Supplementary file 1 [file Table_1.docx]

Supplementary Material

Article Title

Allen Caobi^1^, Jesenia Bonilla^2^, Mario Gomez^3^, Mickensone Andre^1^, Adriana Yndart^1^, Francisco A. Fernandez-Lima^3^, Madhavan P. Nair^1,4^, and Andrea D. Raymond^1^

*** Correspondence:** Andrea D. Raymond, PhD: adraymon@fiu.edu

# Supplementary Data

# Supplementary Figures and Tables

For more information on Supplementary Material and for details on the different file types accepted, please see [here](https://www.frontiersin.org/guidelines/author-guidelines#supplementary-material).

## Supplementary Table 1

| **miRNA** | **Target Gene(s) of Interest:** |
| --- | --- |
| hsa-miR-1290 | MSR1, TNFRSF8 |
| hsa-miR-627-5p | MAP2K4, SEMA3C, PDCD2, SCN3B, COMT, ARHGAP12 |
| hsa-miR-378e | NR2C2, RAB10, KCNIP2, GPR156, PROK2 |
| hsa-miR-150-5p | SV2B, NRCAM, CYP4X1, ADAM19, SNAP23, IRGQ |
| hsa-miR-1246 | GLRB, SLC12A2, SCN3A, OPRM1, TNFRSF8, GPCR |

**Supplementary Table 4.** This table lists the key genes targeted by miRNAs, whose expression has been altered as a result of HIV-1 and/or morphine exposure.
